# Changes in gut microbiota of gallstone mice at different altitudes based on 16S rDNA sequencing

**DOI:** 10.3389/frmbi.2025.1618718

**Published:** 2025-09-04

**Authors:** Song Li, Wenjun Zhu, Runjie Guo, Jinjin Sun, Wei Gao, Shile Wu

**Affiliations:** ^1^ Qinghai Provincial People’s Hospital, Xining, China; ^2^ Department of Clinical Medicine, Qinghai University, Xining, China; ^3^ Qinghai Renji Hospital, Xining, China

**Keywords:** mice, gut microbiota, gallstone, 16s rDNA sequencing, different altitudes (air pressure)

## Abstract

**Objective:**

To establish a gallstone mouse model using a lithogenic diet and investigate changes in the gut microbiota of gallstone mice at different altitudes.

**Methods:**

Sixty mice were randomly assigned to four groups: plain healthy, plain stone, high-altitude healthy, and high-altitude stone. Mice were raised in either plain or high-altitude environments, and a lithogenic diet was used to induce gallstone formation. After 8 weeks, the mice were euthanized, and stone formation was assessed. Blood samples were collected to measure serum total cholesterol (T-CHO), triglycerides (TG), and bile acid (TBA) levels. Fecal samples were also collected for 16S rDNA high-throughput sequencing to analyze the gut microbiota.

**Results:**

TG and T-CHO levels were significantly elevated in gallstone mice in the plain and high-altitude groups. Differential microbiota analysis indicated a decrease in Bacteroidetes and an increase in Firmicutes in the gallstone groups. Several specific bacterial genera showed significant changes in the gallstone mice compared to the healthy controls.

**Conclusion:**

1) Gut microbiota imbalance likely contributes to gallstone formation in mice, and higher microbiota diversity may reduce the incidence of gallstones. 2) The incidence of gallstones is higher at high altitudes than at lower altitudes, possibly due to hypoxic conditions and elevated inflammation levels.

## 1 Introduction

Gallstones are a common benign disease of the biliary system. They are primarily formed through the crystallization or accumulation of a mixture of cholesterol, calcium salts, bile acids, bile pigments, fatty acids, and phospholipids within the gallbladder. Several factors influence gallstone formation, including impaired gallbladder dynamics, abnormal cholesterol metabolism, and abnormal bile acid secretion (Wu et al., 2013). However, the exact pathogenesis remains unclear.The gut microbiota refers to the microbial community within the human gastrointestinal tract, including bacteria, fungi, viruses, and other microorganisms. This dense microbial ecosystem is particularly abundant in areas like the colon and ileum. The gut microbiota encompasses various microorganisms, including anaerobes, aerobes, and actinobacteria, spanning multiple phyla, genera, and species. With approximately 10^14^ bacteria representing over 2,000 species (Caruso et al., 2020), the human gastrointestinal microbiota is highly diverse, playing a crucial role in metabolism and host interactions (Wang et al., 2019).

In recent years, technological advancements and high-throughput sequencing have led to a growing focus on the relationship between gut microbiota imbalance and gallstone formation. Laboratory simulations of bile environments have shown that bacteria can act as nucleating factors, accelerating cholesterol crystallization and increasing gallstone incidence (Sharma et al., 2021). Several studies have also indicated that changes in the gut microbiota are linked to gallstone formation. Dysbiosis may promote the growth of pathogenic bacteria, intensify intestinal and systemic inflammation, and enable the migration of microbiota into the biliary system. This disruption can alter bile composition and the microbiota in the gallbladder, further promoting gallstone formation (Tedesco et al., 2018).

The Qinghai-Tibet Plateau, known as the “Roof of the World,” has an average altitude of 4,000 meters, with oxygen levels about 50%-60% of those at sea level. The low-pressure hypoxic environment is a defining feature of the plateau. Globally, the incidence of gallstones is approximately 10%-20%, with higher prevalence in the United States and European countries, particularly among middle-aged and elderly populations (Lammert et al., 2016). In China, the incidence has been rising steadily due to improved living standards and changes in diet, reaching nearly 11% (Su et al., 2020). The prevalence of gallstones in the Qinghai-Tibet Plateau is significantly higher than the national average, with some studies reporting rates of 15%-20%. This may be linked to the high-altitude, low-oxygen environment and unique dietary habits (Yang et al., 2018; Ma et al., 2022). A study by Zhang et al. (2018) found significant alterations in the gut microbiota of mice at high altitudes compared to those in plain regions. However, little research has explored whether these changes in gut microbiota contribute to the development of gallstones under high-altitude conditions. This study uses 16S rDNA high-throughput sequencing to analyze the fecal microbiota of mice in different groups, aiming to investigate the relationship between gut microbiota changes and gallstone formation at high altitudes and provide theoretical insights for gallstone prevention.

## 2 Materials and methods

### 2.1 Instruments and reagents

Fecal DNA extraction kit (Meiji Bio Co.); Trans2K DNA Marker and agarose (Beijing Quanshi Jin Co.); nucleic acid dye (Shanghai Shenggong Co.); AxyPrep DNA gel recovery kit (AXYGEN); lithogenic diet (Medison Co.); tabletop centrifuge (Shanghai Anting Scientific Instrument Co.); vortex mixer (Haimen Qilin Bell Instrument Manufacturing Co., Ltd.); dry constant temperature incubator (Hangzhou Meisheng Instrument Co., Ltd.); nucleic acid protein quantification instrument (Hangzhou Aokang); electrophoresis power supply and tank (Beijing LiuYi Instrument Factory); gel imaging system (Shanghai Tianneng Technology Co., Ltd.); NanoDrop micro-spectrophotometer (Thermo Fisher Scientific); ABI GeneAmp PCR instrument (ABI); Quantus fluorometer (Promega); Illumina sequencing instrument (Illumina); QuantiFluor™-ST Blue Fluorescent Quantification System (Promega). Total cholesterol, triglyceride, and bile acid assay kits (Nanjing Jianchen Co.); lithogenic diet (Medison Co.).

### 2.2 Experimental animals and grouping

Sixty adult male C57BL/6J mice (6–8 weeks old) were randomly assigned to four groups:

Plain Healthy Group: 10 mice were raised at an altitude of 1000m in Urumqi under pathogen-free conditions and fed a regular diet.

Plain Stone Group: 20 mice, raised at 1000m and fed a lithogenic diet (1.25% cholesterol and 0.5% bile acid) to induce gallstones.

High-altitude Healthy Group: 10 mice, raised at 4500m (simulated hypoxic environment in a low-pressure chamber) and fed a regular diet.

High-altitude Stone Group: 20 mice were first acclimated for one week in the high-altitude environment(4500m), then fed a lithogenic diet under the same conditions to induce gallstone formation.

All mice were raised for 8 weeks and then euthanized. After euthanizing the mice in the stone groups, the gallbladders were dissected and visually examined. Mice with clearly visible cholesterol gallstones in the gallbladder were identified as gallstone-positive samples. Among them, 14 mice in the plain gallstone group and 15 mice in the high-altitude gallstone group were confirmed to have gallstones. Fecal samples were then randomly collected from 10 gallstone-positive mice in each group. The animal study was approved by the Animal Care Committee of Qinghai Provincial People’s Hospital, Qinghai University School of Medicine (Approval No. QRH-2020-0014), and was conducted in accordance with local regulations and institutional guidelines.

### 2.3 Experimental methods

#### 2.3.1 Laboratory indicator testing and statistical analysis

After ether anesthesia and euthanasia of the mice, blood samples were collected from the fundus venous plexus of the eye. The collected blood samples were divided into four groups based on the above groupings and whether gallstones were formed. Each group contained 10 samples: Plain Healthy Group (FB-PK), Plain Stone Group (FB-PM), High-altitude Healthy Group (FB-GK), and High-altitude Stone Group (FB-GM). The blood samples were centrifuged at 3000 rpm for 5 minutes, and the serum was stored at -80°C for further analysis. Biochemical kits were used to measure the levels of total cholesterol (T-CHO), triglycerides (TG), and total bile acids (TBA) in the serum of each group.

#### 2.3.2 16S rDNA high-throughput sequencing method

##### 2.3.2.1 Sample collection

Fecal samples were collected one day before euthanasia and divided into four groups, each containing 10 samples, as described earlier. The samples were placed in sterilized fecal collection containers and stored at -80°C for preservation within 6 hours.

##### 2.3.2.2 Genomic DNA extraction

Genomic DNA was extracted using the Tiangen DNA extraction kit (DP328). 200 mg of the sample was subjected to lysis, column purification, and washing steps, followed by elution using 50 μL of elution buffer (TB). The quality of the extracted genomic DNA was assessed using 1% agarose gel electrophoresis, and DNA content was measured using a NanoDrop micro-spectrophotometer.

##### 2.3.2.3 PCR amplification

The V3–V4 hypervariable regions of the bacterial 16S rRNA gene were amplified using primers 341F (5′-CCTACGGGNGGCWGCAG-3′) and 806R (5′-GACTACHVGGGTATCTAATCC-3′) (Caporaso et al., 2011; Klindworth et al., 2013). Each sample was replicated three times. The PCR products from the same sample were pooled and detected by 2% agarose gel electrophoresis. The PCR products were purified using the AxyPrep DNA gel recovery kit (AXYGEN), and the gel was cut and recovered. The products were eluted with Tris-HCl, and 2% agarose gel electrophoresis was used for detection.

##### 2.3.2.4 Fluorescence quantification

Based on preliminary electrophoresis quantification results, the PCR products were quantified using the QuantiFluor™-ST Blue Fluorescent Quantification System. After quantification, the products were mixed in the appropriate proportions according to the sequencing requirements for each sample.

##### 2.3.2.5 Illumina PE250 library construction

Y-shaped adapters were ligated to the DNA fragments. Magnetic beads were used to remove adapter self-ligation products. PCR amplification was performed to enrich the library templates. Sodium hydroxide denaturation was applied to generate single-stranded DNA fragments.

##### 2.3.2.6 Illumina PE250 sequencing

One end of the DNA fragments was complementary to the primer and fixed on the chip. The other end was randomly complementary to another nearby primer, forming a “bridge.” PCR amplification generated DNA clusters, which were then linearized to single strands. Modified DNA polymerase and fluorescently labelled dNTPs were used in each cycle to synthesize one base at a time. Laser scanning was used to read the nucleotide type added during the first cycle. Fluorescent and terminator groups were chemically cleaved, restoring the 3’ end and allowing continued polymerization of the second nucleotide. Fluorescent signals from each round were collected to determine the sequence of the template DNA fragments.

### 2.4 Statistical analysis

#### 2.4.1 Laboratory indicator statistical analysis

Group comparisons were performed using SPSS 22.0 software for statistical analysis. Normally distributed and variance-homogeneous data were expressed as (x ± s), and paired t-tests were used for statistical analysis. Non-normally distributed data were presented as median (P25, P75) and analyzed by rank-sum test. A P value of <0.05 was considered statistically significant.

#### 2.4.2 Gut microbiota analysis

##### 2.4.2.1 Data processing and statistical analysis

The raw data sequences were processed using Trimmomatic (version 0.35) software. The sliding window method was used to scan the sequences, and bases with an average quality score below 20 were trimmed. Sequences shorter than 50bp were also removed. Flash (version 1.2.11) software was used to merge the qualified paired-end raw data with a maximum overlap of 200bp to obtain complete paired-end sequences. The clean tags were obtained after filtering sequences with “N” bases, sequences with more than eight repeated bases, and sequences shorter than 200bp using QIIME’s split_libraries (version 1.9.0). Research software was used to remove chimeric sequences, and valid tags were used for OTU classification. A summary file was generated for the entire quality control process.

##### 2.4.2.2 OTU classification and annotation

Vsearch (version 2.4.2) and the uclust_ref method in QIIME 1.9.0 were meticulously employed to classify the clean sequences into OTUs with a stringent 97% similarity threshold. The most abundant sequence in each OTU was carefully chosen as the representative sequence. The RDP classifier Naive Bayesian classification algorithm was then used to compare the representative sequences with the database for annotation, ensuring the precision of our OTU annotation process.

##### 2.4.2.3 α Diversity analysis

α diversity was calculated using the Qiime software (version 1.9.1) with Shannon, Simpson, and Chao1 indices. Rarefaction, species-level, and species accumulation curves were plotted using R software (version 2.15.3), and differences in α diversity indices between groups were analyzed using T-test and Wilcoxon tests.

##### 2.4.2.4 β Diversity analysis

β diversity was calculated using Qiime software (version 1.9.1) based on Unifrac distance, and a UPGMA tree was constructed. PCoA and NMDS plots were generated using R software (version 2.15.3) with the WGCNA, stats, and ggplot2 packages for PCoA and the vegan package for NMDS. Differences in β diversity indices were analyzed using T-test and Wilcoxon tests in R software.

##### 2.4.2.5 Differential species analysis between groups:

To identify the species that differ between groups at various taxonomic levels (Phylum, Class, Order, Family, Genus, Species), we used R software to conduct t-tests and create visual plots. Species with significant differences (P < 0.05) were identified.

## 3 Results

### 3.1 Laboratory indicator results

By measuring serum triglycerides (TG), total cholesterol (T-CHO), and total bile acids (TBA) in mice, it was determined that T-CHO data followed a normal distribution with homogeneity of variance (p = 0.763); therefore, paired t-tests were used for intergroup comparisons. In contrast, TG and TBA data did not follow a normal distribution and showed heterogeneity of variance (p = 0.003 and p = 0.005, respectively), so non-parametric rank-sum tests were used instead. As shown in **Table 1**, it was found that under normal altitude conditions, mice in the FB-PM group had significantly higher TG and T-CHO levels compared to those in the FB-PK group (H = -17.300, p = 0.006; t = -11.340, p = 0.001). Similarly, at high altitudes, TG and T-CHO levels were significantly higher in the FB-GM group than in the FB-GK group (H = -16.200, p = 0.012; t = -14.515, p = 0.001). However, when comparing different altitude conditions, no significant differences in biochemical indicators were observed between the FB-PK and FB-GK groups or between the FB-PM and FB-GM groups.

**Table 1:** Statistical table of serum biochemical indicator levels in each group (N=10).

Group	TG (mmol/L)	T-CHO (mmol/L)	TBA (μmol/L)
FB-PK	1.315 (0.970, 2.441)	5.012 ± 2.270	23.531 (19.782, 25.252)
FB-PM	4.885 (3.603, 9.237)^a^	18.593 ± 3.033^a^	28.622 (25.253, 30.722)
FB-GK	1.625 (0.938, 3.038)^b^	6.597 ± 2.152^b^	24.415 (15.150, 34.933)
FB-GM	4.760 (4.100, 5.392)^ac^	21.357 ± 2.389^abc^	31.985 (24.830, 48.403)^a^

^a^indicates P < 0.05 compared with the Plain Healthy Group (FB-PK); ^b^indicates P < 0.05 compared with the Plain Gallstone Group (FB-PM); ^c^indicates P < 0.05 compared with the High-altitude Healthy Group (FB-GK).

### 3.2 Quality assessment of 16S rDNA sequencing data

#### 3.2.1 Sequencing quality analysis

A total of 40 samples were analyzed, with clean tag counts ranging from 39,179 to 79,861 and valid tag counts ranging from 33,416 to 63,366. In all samples, the proportion of valid tags exceeded 70%. The shortest valid nucleotide length ranged from 201 to 228 bp, while the longest ranged from 466 to 490 bp.

The number of observed OTUs (Operational Taxonomic Units) in each group was as follows: Plain-region mice without gallstones: 6,690 OTUs; plain-region mice with gallstones: 3,189 OTUs; High-altitude mice without gallstones: 6,268 OTUs; high-altitude mice with gallstones: 3,945 OTUs.

#### 3.2.2 Rarefaction curve

**Figure 1:**
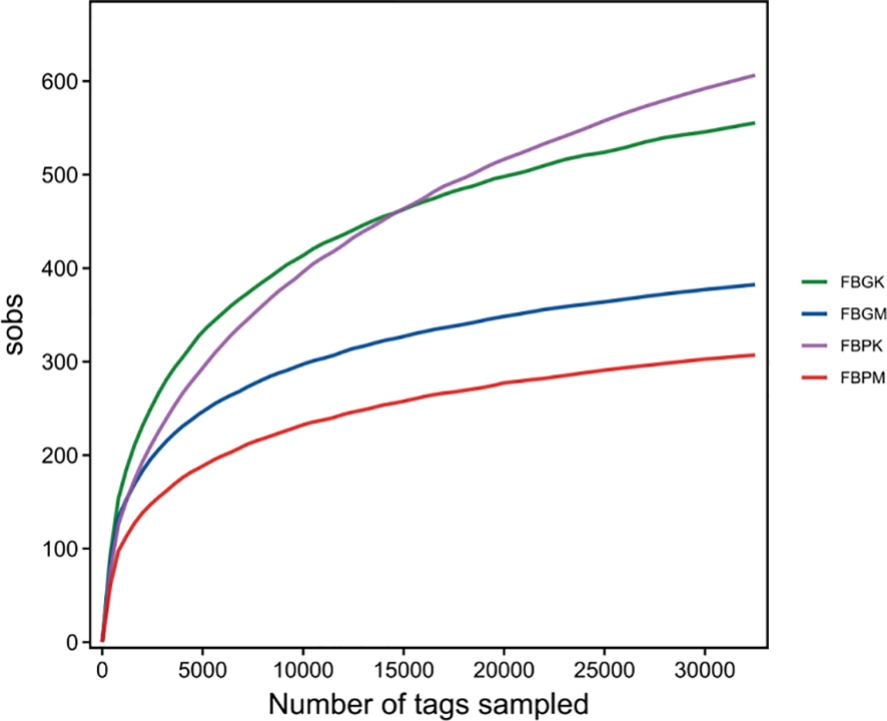
Rarefaction curves of gut microbiota in the four groups of mice.

Rarefaction curves were generated for the Plain Healthy Group (FB-PK), Plain Stone Group (FB-PM), High-altitude Healthy Group (FB-GK), and High-altitude Stone Group (FB-GM) (see **Figure 1**). As the sampling depth increased, the discovery rate of new species gradually declined, indicating that the sequencing depth was sufficient for each group.

### 3.3 Species diversity analysis

#### 3.3.1 Alpha diversity analysis

Alpha diversity analysis is commonly used to assess microbial community diversity within a single sample. **Table 2** shows a significant difference in microbial abundance and the Chao1 index between the FB-PK (Plain Healthy) and FB-PM (Plain Stone) groups. The FB-PM group exhibited fewer detected species, indicating that, under normal altitude conditions, the gut microbiota of healthy mice had greater diversity when considering only species composition.

**Table 2:** Alpha diversity analysis of gut microbiota in four groups (N=10).

Group	Observed species	chao1	shannon	simpson
FB-PK	669 ± 37.89	816.38 ± 28.19	5.43 ± 0.55	0.92 ± 0.05
FB-PM	318.9 ± 70.30^a^	366.44 ± 73.60^a^	4.93 ± 0.57	0.91 ± 0.03
FB-GK	626.8 ± 31.67^a^	724.03 ± 27.63^a^	5.55 ± 0.74	0.90 ± 0.06
FB-GM	394.5 ± 33.64^bc^	443.54 ± 34.49^bc^	5.77 ± 0.80^b^	0.94 ± 0.038^b^

^a^indicates P < 0.05 compared with the Plain Healthy Group (FB-PK); ^b^indicates P < 0.05 compared with the Plain Gallstone Group (FB-PM); ^c^indicates P < 0.05 compared with the High-altitude Healthy Group (FB-GK).

When comparing the FB-PK (Plain Healthy) and FB-GK (High-altitude Healthy) groups, significant differences in gut microbiota species richness were observed between high-altitude and plain-region healthy mice, with the latter showing higher diversity. The Chao1 index further confirmed that plain-region mice had a richer gut microbiota.

The Chao1 index was significantly lower in the FB-GM (High-altitude Stone) group than in the FB-GK (High-altitude Healthy) group. When considering species richness alone, high-altitude healthy mice exhibited greater microbial diversity. However, no significant difference was found between the two groups when also accounting for microbial abundance.

Finally, comparisons between the FB-GM (High-altitude Stone) and FB-PM (Plain Stone) groups revealed statistically significant differences in the Observed Species index, Chao1 index, Shannon index, and Simpson index. It suggests that the gut microbiota diversity in high-altitude gallstone mice was higher than that in plain-region gallstone mice.

#### 3.3.2 Species richness rank curve

**Figure 2:**
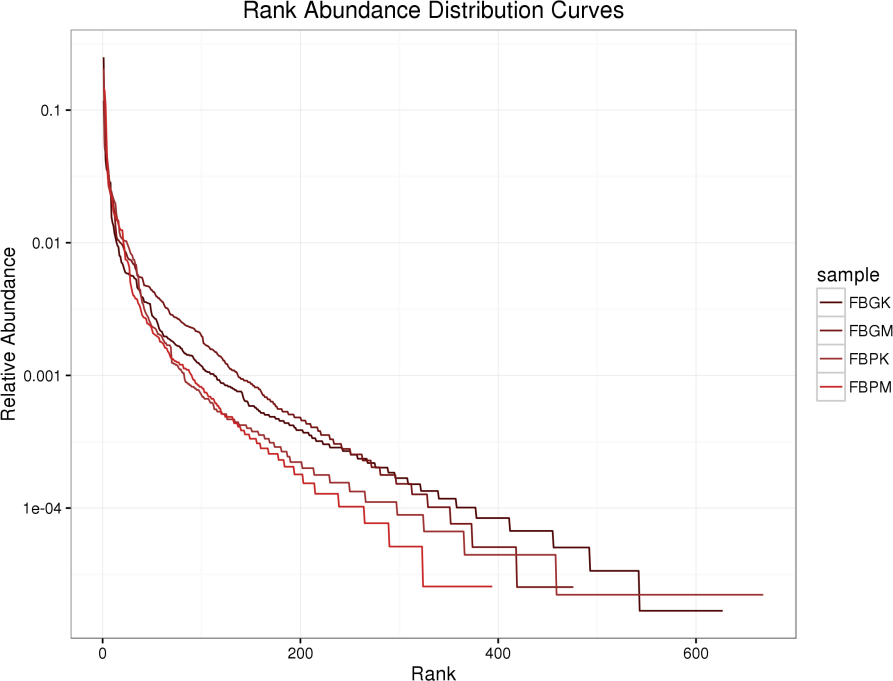
Species richness rank curve.

Species richness rank curves were generated for the FB-PK (Plain Healthy), FB-PM (Plain Stone), FB-GK (High-altitude Healthy), and FB-GM (High-altitude Stone) groups. As shown in **Figure 2**, gut microbiota diversity ranked from highest to lowest as follows: FB-PK > FB-GK > FB-GM > FB-PM. Based on species count, microbial diversity was higher in normal-altitude regions than in the high-altitude areas.

#### 3.3.3 Beta diversity analysis

**Figure 3:**
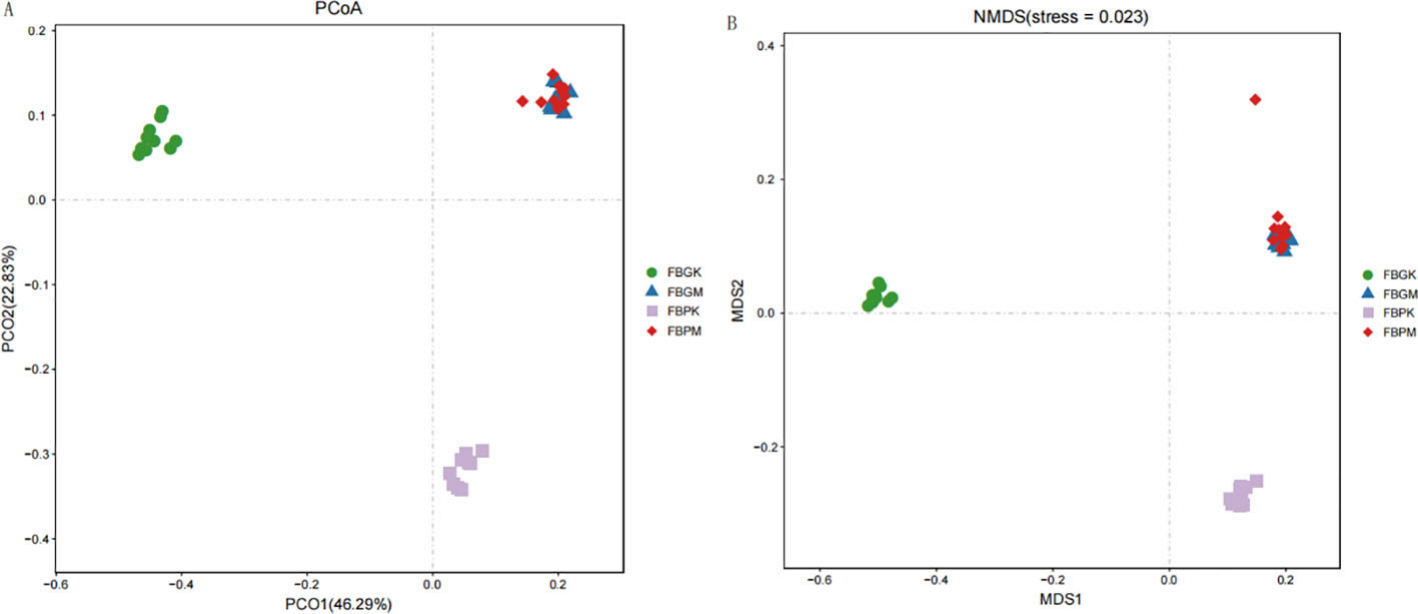
Beta diversity analysis of gut microbiota in the four groups of mice. **(A)** PCoA Analysis of Gut Microbiota; **(B)** NMDS Analysis of Gut Microbiota.

To compare the gut microbiota of the four groups, a principal coordinate analysis (PCoA) plot and a Non-metric Multidimensional Scaling (NMDS) plot was generated based on Unweighted UniFrac distance (**Figure 3**).

The figures indicate that differences between samples and microbial communities among the groups were relatively small(*p*=0.001). However, the FB-PM (Plain Stone) and FB-GM (High-altitude Stone) groups exhibited microbial compositions that were more similar to those of the other groups. This suggests that the gut microbiota composition of gallstone-afflicted mice at high altitudes resembles those at typical altitudes.

### 3.4 Analysis of differential gut microbiota

#### 3.4.1 Phylum-level analysis

**Figure 4:**
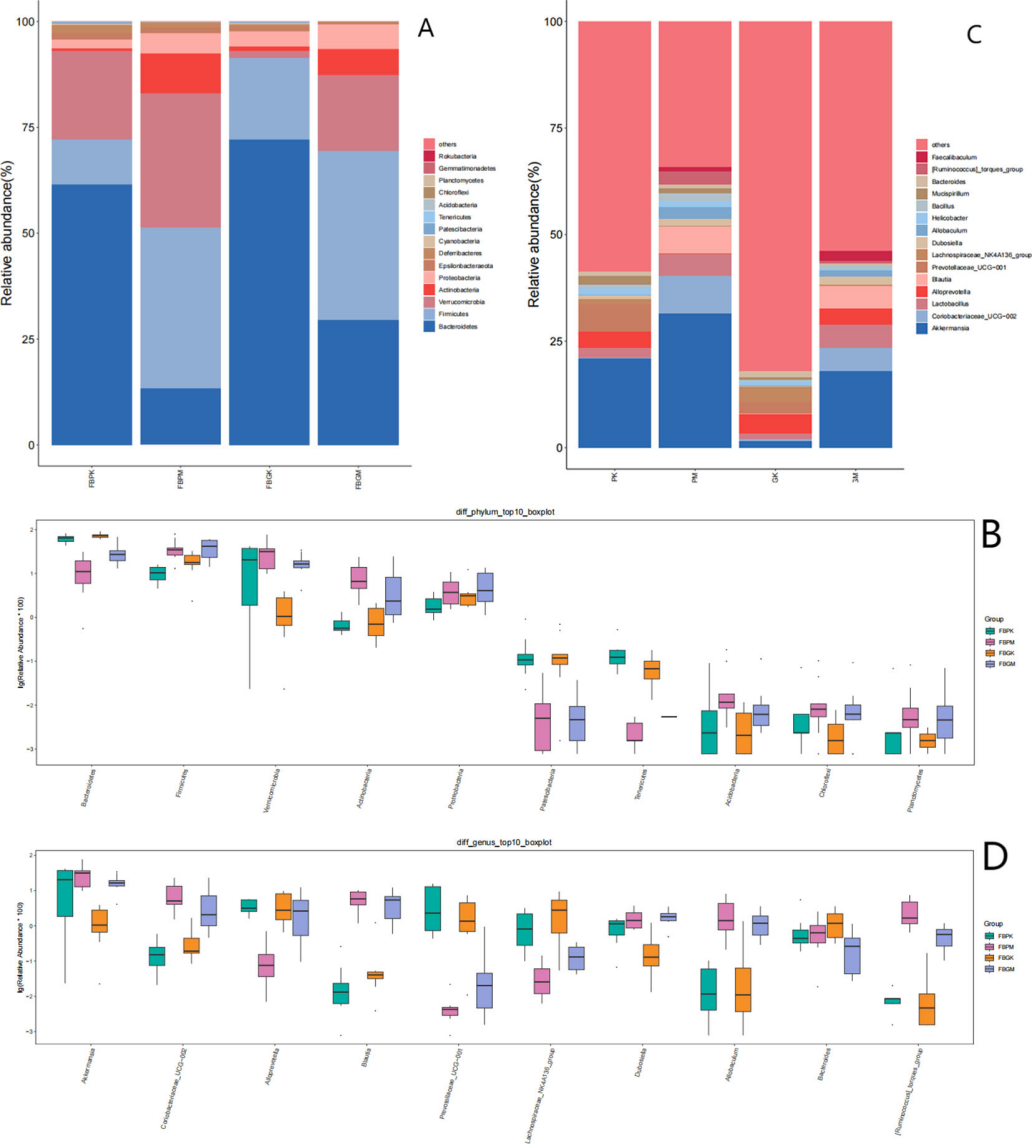
Comparison of gut microbiota among four groups of mice. **(A)** Bar Chart of Relative Abundance of Gut Microbiota at the Phylum Level in the Four Groups of Mice. **(B)** Boxplot of Differential Gut Microbiota at the Phylum Level in the Four Groups of Mice. **(C)** Bar Chart of Relative Abundance of Gut Microbiota at the Genus Level in the Four Groups of Mice. **(D)** Boxplot of Differential Gut Microbiota at the Genus Level in the Four Groups of Mice.

The operational taxonomic units (OTUs) of gut microbiota in the four groups (FB-PK, FB-PM, FB-GK, and FB-GM) were analyzed, with results in **Figures 4A, B**. The six most abundant phyla across all groups were: Bacteroidetes, Firmicutes, Verrucomicrobia, Actinobacteria, Proteobacteria, Epsilonbacteraeota.

In the FB-PM (Plain Stone) and FB-GM (High-altitude Stone) groups, the relative abundance of Bacteroidetes and Patescibacteria was significantly lower compared to the healthy groups. At the same time, Firmicutes showed a notable increase in relative abundance. Additionally, the FB-GK (High-altitude Healthy) group exhibited a significant reduction in Verrucomicrobia compared to the other groups.

#### 3.4.2 Genus-level analysis

The relative abundance of bacterial genera in the four groups was analyzed, with results shown in **Figure 4C**:FB-PK (Plain Healthy) group: Akkermansia, Lactobacillus, Alloprevotella, and Prevotellaceae_UCG-001 were the top four most abundant genera. FB-GK (High-altitude Healthy) group: The top four most abundant genera were Alloprevotella, Lachnospiraceae_NK4A136_group, Prevotellaceae_UCG-001, and Akkermansia. FB-PM (Plain Stone) and FB-GM (High-altitude Stone) groups: The top four most abundant genera were Akkermansia, Coriobacteriaceae_UCG-002, Lactobacillus, and Blautia.

Intergroup comparisons of bacterial genera were conducted using the Kruskal-Wallis test. The results presented in **Figure 4D** show that the abundance of Akkermansia in the FB-GK group was significantly lower than in the other three groups.Coriobacteriaceae_UCG-002, Allobaculum, Blautia, and [Ruminococcus]_torques_group were significantly more abundant in the FB-PM and FB-GM groups than in the healthy groups. Conversely, Prevotellaceae_UCG-001 and Lachnospiraceae_NK4A136_group were significantly less abundant in the FB-PM and FB-GM groups compared to the healthy groups.

### 3.5 LEfSe multilevel differential taxa analysis

**Figure 5:**
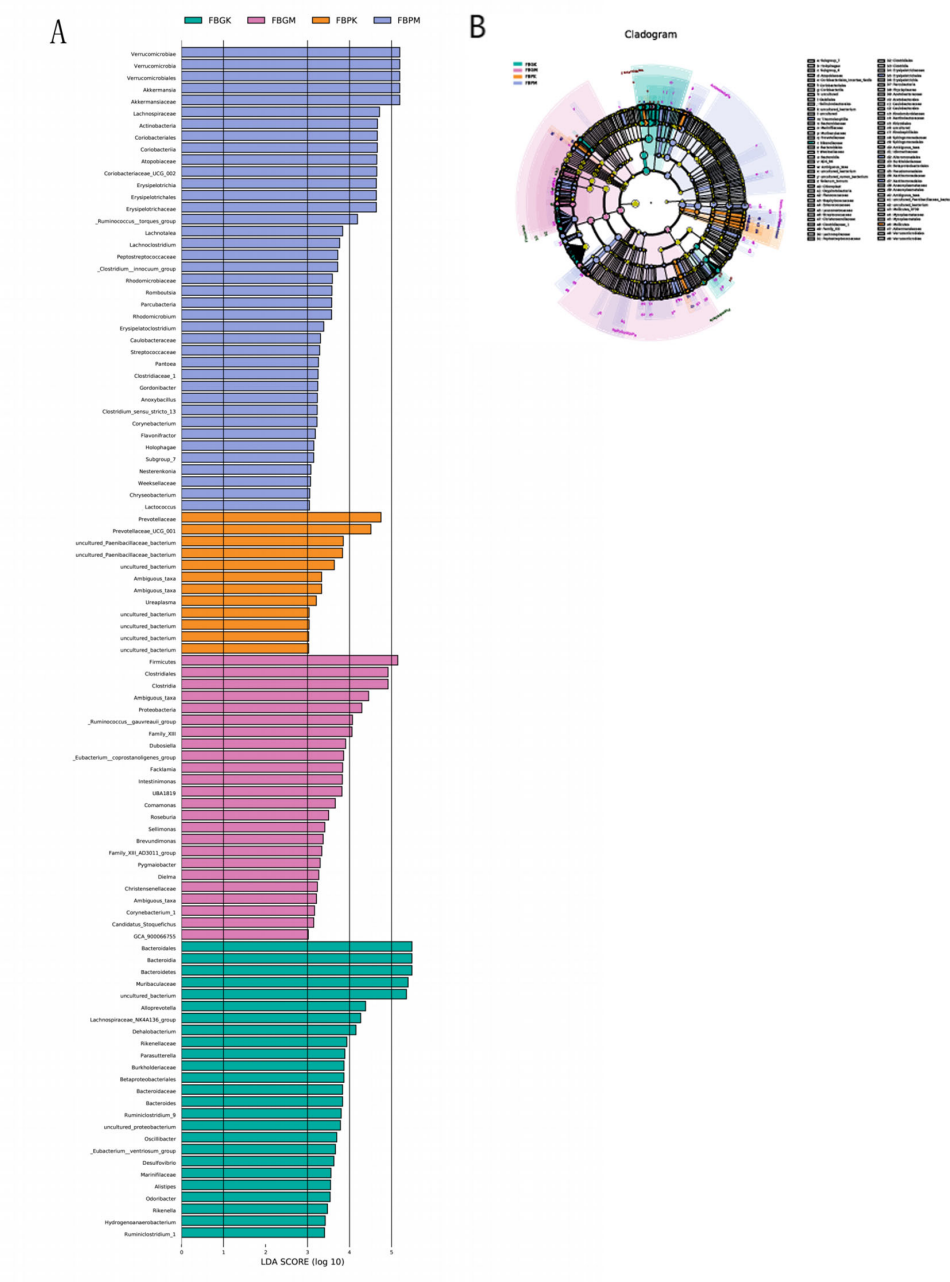
LEfSe multilevel differential analysis of gut microbiota among the four groups of mice. **(A)** Differential taxa contribution analysis **(B)** Differential taxa annotation analysis.

Differential taxa contribution analysis of the gut microbiota among the four groups of mice was performed using LEfSe, with an LDA score >3 considered as a significant difference (see **Figure 5** and **Table 3**). The results revealed distinct differences in gut microbial composition between the groups. The FB-PK group was significantly enriched in taxa belonging to the phylum Actinobacteria and its related clades. In the FB-PM group, differential taxa were predominantly enriched in the phylum Verrucomicrobia and its subordinate taxa. Both the FB-GK and FB-GM groups showed enrichment of taxa within the phylum Firmicutes. Specifically, the FB-GK group was enriched in genera associated with the families Lachnospiraceae and Ruminococcaceae, while the FB-GM group displayed a relative increase in facultative anaerobic bacteria.

**Table 3:** Classification of genus-level LEfSe multilevel differential analysis results of gut microbiota among the four groups of mice (N=10).

Phylum group	FB-PK	FB-PM	FB-GK	FB-GM
Verrucomicrobia		Akkermansia Akkermansiaceae Verrucomicrobiales Verrucomicrobiae		
Bacteroidetes	Prevotellaceae Prevotellaceae_UCG_001		Rikenella Odoribacter Alistipes Bacteroides Bacteroidaceae Rikenellaceae Alloprevotella Muribaculaceae	
Firmicutes		Ruminococcus_gnavus_group Lactococcus Flavonifractor Corynebacterium Clostridium_sensu_stricto_13 Anoxybacillus Gordonibacter Clostridiaceae_1 Erysipelatoclostridium Romboutsia Clostridium_innocuum_group Peptostreptococcaceae Lachnoclostridium Lachnotalea Ruminococcus_torques_group Erysipelotrichaceae Erysipelotrichales Erysipelotrichales Leuconostoc Streptococcaceae	Christensenellaceae_R7_group Ruminococcaceae_UCG_010 Lachnospira Ruminiclostridium_1 Hydrogenoanaerobacterium Oscillibacter Ruminiclostridium_9 Lachnospiraceae_NK4A136_group Christensenellaceae Eubacterium_ventriosum_group	Christensenellaceae Family_XIII_AD3011_group Sellimonas Roseburia Intestinimonas Dubosiella Family_XIII Ruminococcus_gauvreauii_group Clostridia Clostridiales Corynebacterium_1 Dielma Pygmaiobacter
Proteobacteria			Desulfovibrio Betaproteobacteriales Burkholderiaceae Parasutterella	Comamonas
Actinobacteria	Mollicutes Ureaplasma	Coriobacteriaceae_UCG_002 Atopobiaceae Coriobacteriia Coriobacteriales		

## 4 Discussion

Gallstones are a common condition in general surgery, with an incidence rate of 10% or higher. The disease follows the well-known “4F” characteristics, being more prevalent in females, obese individuals, those with multiple pregnancies, and individuals over 40 years old. With changes in dietary habits and lifestyle, the proportion of gallstones—particularly cholesterol gallstones—has been increasing annually (Jayasoma et al., 2022). Studies suggest that the risk of gallstones is significantly higher in high-altitude regions compared to plain regions. It may be attributed to environmental factors such as cold temperatures, low oxygen levels, increased consumption of high-fat and high-sugar foods, irregular sleep patterns, and reduced physical activity (Rosa et al., 2020). Gallstone formation is a multifactorial process. Current research indicates that it is primarily associated with bile supersaturation, nucleation processes, and dietary factors, which collectively promote cholesterol crystallization and subsequent gallstone development. Additionally, recent studies (Carey, 1993; Dowling, 2000; Venneman and van Erpecum, 2010) have confirmed that bacteria can be detected in most cholesterol gallstones. Bacteria may contribute to gallstone formation by triggering gallbladder inflammation, promoting immunoglobulin synthesis and secretion, and acting as nucleating factors upon bacterial cell death, accelerating the crystallization process (DeLeon et al., 2020).

The gut microbiota and the human body exist in a symbiotic relationship, where both depend on each other and interact to maintain homeostasis. Recent studies have highlighted the crucial role of gut microbiota in aiding digestion, influencing metabolic diseases, and regulating intestinal immunity (Lin and Zhang, 2017; Cox et al., 2022). The gut microbiota establishes a symbiotic relationship with the host, producing hundreds of proteins and metabolic products. These metabolites regulate various key functions of the host, including nutrient processing, energy homeostasis, and immune system development (Martin-Gallausiaux et al., 2021).

Animal experiments allow for controlled variables such as diet and genetic background, enabling the isolated study of altitude’s effects on gut microbiota and its relationship with gallstone formation. Alpha diversity analysis revealed that high-altitude mice had lower species richness than normal-altitude mice. However, overall diversity remained similar between the two groups when considering species abundance. It suggests that while altitude influences gut microbiota composition, its effect on dominant bacterial populations may be relatively minor. When comparing two groups of mice with gallstones, those at high altitudes exhibited greater gut microbiota diversity. It may be related to the persistent hypoxic environment and lithogenic diet associated with high altitudes, which could slow the decline in microbial diversity under disease conditions. However, this hypothesis currently lacks theoretical support and warrants further investigation. Across altitude groups, healthy mice consistently showed higher gut microbiota diversity than those with gallstones, supporting the idea that greater microbial diversity may reduce the risk of gallstone formation—an observation consistent with previous studies (Fremont-Rahl et al., 2013; Wang et al., 2017). Beta diversity analysis further revealed that the gut microbiota of gallstone-bearing mice clustered more tightly under both high- and low-altitude conditions. In contrast, that of healthy mice was more dispersed. This pattern mirrors the changes observed in the gut microbiota of humans with gallstones, further validating the similarity in gut microbiota associated with gallstone disease (Wang et al., 2020).

Phylum-level analysis of gut microbiota across the four groups revealed that Bacteroidetes, Firmicutes, Verrucomicrobia, and Actinobacteria were the dominant phyla, with Bacteroidetes and Firmicutes comprising the most significant proportions. A study by Grigor’eva (2021) identified the Firmicutes/Bacteroidetes ratio as a key marker in gut microbiota composition and its correlation with obesity and gallstones. Their findings indicated that gallstone patients exhibited a decrease in Bacteroidetes and an increase in Firmicutes, which may disrupt bile acid metabolism and elevate the risk of cholesterol gallstone formation. Additionally, reducing Bacteroidetes may lower the production of secondary bile acids, altering bile composition and further promoting gallstone formation (Ding et al., 2023). In this experiment, the abundance of Bacteroidetes in the gut microbiota of both gallstone groups was significantly lower than that in healthy mice. In comparison, the abundance of Firmicutes was notably higher. These findings are broadly consistent with those reported by Grigor’eva et al. (2021) in their study on the gut microbiota of patients with cholelithiasis. Altitude had a relatively minor impact on gut microbiota composition in gallstone mice. No significant differences between the two gallstone groups were observed in Firmicutes and Bacteroidetes. However, differences were noted in Proteobacteria and Actinobacteria, though their abundance remained below 1%, suggesting that the gut microbiota composition of gallstone mice was broadly similar across altitudes. Based on the results of LEfSe multilevel species difference analysis, the differential gut microbiota between the FB-PM and FB-GM gallstone groups and healthy mice were primarily concentrated among facultative anaerobes within the Verrucomicrobiota and Firmicutes phyla. An increase in facultative anaerobes is often regarded as a hallmark of gut microbiota dysbiosis (Rigottier-Gois, 2013; Kriss et al., 2018), suggesting that the onset and progression of gallstone disease may be closely associated with microbial imbalances in the gut.

At the genus level, Akkermansia abundance in the high-altitude healthy group (FB-GK) was significantly lower than in the plain healthy group (FB-PK). Akkermansia muciniphila, a mucin-degrading bacterium, is crucial in maintaining intestinal barrier integrity and preventing inflammatory diseases. It is often negatively correlated with disease development. In hypoxic high-altitude environments, reduced oxygen levels may lead to a decline in Akkermansia muciniphila, weakening the gastrointestinal barrier (Mishra and Bakshi, 2023). The significant decrease in Akkermansia observed in this study may be a direct consequence of hypoxic conditions. Allobaculum, a bacterial genus commonly found in the mouse gut, showed a significant increase in both gallstone groups (FB-PM and FB-GM), likely due to long-term consumption of the lithogenic diet (Zheng et al., 2021). The [Ruminococcus]_torques_group, part of the Ruminococcus family, has been identified in bile and gallstones of gallstone patients through ITS and 16S rDNA sequencing, as reported by Hu et al. (2023). Additionally, a bidirectional Mendelian randomization study found that an increase in the genus UCG003, also from the Ruminococcus family, was positively correlated with a higher risk of cholecystitis (OR = 1.25, P = 0.04) (Miao et al., 2023). This finding aligns with our experimental results.

By comparing triglyceride (TG), total cholesterol (T-CHO), and total bile acid (TBA) levels in mouse serum, gallstone mice were found to have higher total cholesterol and bile acid levels than non-gallstone mice. Elevated serum cholesterol can stimulate the expression of the ATP-binding cassette transporter G5/8 (ABCG5/8), which is primarily found in hepatic cells, bile ducts, and intestinal epithelial cells. ABCG5/8 facilitates cholesterol transport from serum into bile for excretion, a process that can promote gallstone formation (Sun et al., 2022). Consequently, gallstone mice exhibited higher cholesterol levels in bile than non-gallstone mice. Bile acids are cholesterol-derived metabolic products synthesized in the liver via 7α-hydroxylase activity (Hubacek and Bobkova, 2006). An imbalance between bile acids and cholesterol is a key factor in cholesterol gallstone formation. In this study, gallstone mice had increased bile acid concentrations, potentially due to higher serum cholesterol levels, which provided more substrate for bile acid synthesis. Besides, Rodent-specific bile acid metabolism – unlike humans, rodents can hydroxylate secondary bile acids, maintaining their hydrophilicity and increasing the bile acid pool (Chiang, 2017). This distinction may explain higher bile acid concentrations in rodent bile. Moreover, Variability in bile acid types, with their specific roles in gallstone formation, requires further validation. When analyzing inflammatory mediators, gallstone mice showed significantly elevated serum inflammatory markers, suggesting an inflammatory response linked to gallstone formation. However, no significant differences were found in bile inflammatory mediators between groups. The relationship between inflammatory markers and gallstones remains inconclusive. Some studies suggest that inflammatory mediators like LPS, TNF, and IL-1 may inhibit 7α-hydroxylase mRNA transcription, altering bile acid levels and increasing gallstone risk (Feingold et al., 2013). Future research should consider larger sample sizes or human bile sample analysis to explore this correlation further.

In summary, gut microbiota imbalance may play a crucial role in gallstone formation in mice. A diverse gut microbiota helps maintain bile acid metabolism balance and reduces gallstone incidence. In gallstone mice, Bacteroidetes abundance decreased while Firmicutes increased, disrupting the Bacteroidetes-to-Firmicutes ratio. This imbalance may alter bile acid metabolism, change bile composition, and promote cholesterol crystallization. Gallstone incidence is higher at high altitudes than in lowland regions, likely due to hypoxia, high-fat/high-sugar diets, irregular sleep patterns, and elevated inflammation levels. This study found that Akkermansia was significantly reduced in high-altitude mice, which may weaken intestinal barrier integrity and increase gallstone risk. Future research should explore the interactions between gut microbiota, bile acid metabolism, and inflammation regulation to uncover the microbiological mechanisms underlying gallstone formation. Targeted gut microbiota modulation could offer new insights into personalized prevention and treatment strategies for gallstones.

## Data Availability

The original contributions presented in the study are publicly available. This data can be found here: NCBI BioProject, accession PRJNA1313303.
